# Tunable Electronic Properties of Type-II SiS_2_/WSe_2_ Hetero-Bilayers

**DOI:** 10.3390/nano10102037

**Published:** 2020-10-15

**Authors:** Yue Guan, Xiaodan Li, Ruixia Niu, Ningxia Zhang, Taotao Hu, Liyao Zhang

**Affiliations:** 1 College of Science, University of Shanghai for Science and Technology, Shanghai 200093, China; 182282018@st.usst.edu.cn (Y.G.); 182282006@st.usst.edu.cn (R.N.); 182282021@st.usst.edu.cn (N.Z.); lyzhang@usst.edu.cn (L.Z.); 2 School of Physics, Northeast Normal University, Changchun 130024, China; hutt262@nenu.edu.cn

**Keywords:** first principle, hetero-bilayer, type-II band alignment, tunable band gap

## Abstract

First-principle calculations based on the density functional theory (DFT) are implemented to study the structural and electronic properties of the SiS_2_/WSe_2_ hetero-bilayers. It is found that the AB-2 stacking model is most stable among all the six SiS_2_/WSe_2_ heterostructures considered in this work. The AB-2 stacking SiS_2_/WSe_2_ hetero-bilayer possesses a type-II band alignment with a narrow indirect band gap (0.154 eV and 0.738 eV obtained by GGA-PBE and HSE06, respectively), which can effectively separate the photogenerated electron–hole pairs and prevent the recombination of the electron–hole pairs. Our results revealed that the band gap can be tuned effectively within the range of elastic deformation (biaxial strain range from −7% to 7%) while maintaining the type-II band alignment. Furthermore, due to the effective regulation of interlayer charge transfer, the band gap along with the band offset of the SiS_2_/WSe_2_ heterostructure can also be modulated effectively by applying a vertical external electric field. Our results offer interesting alternatives for the engineering of two-dimensional material-based optoelectronic nanodevices.

## 1. Introduction

In the past two decades, the emergence of two-dimensional layered materials [1,2,3,4] has attracted tremendous attention of researchers due to their novel electronic properties, such as high carrier mobility [5,6], high thermal conductivity [7] and excellent on/off ratio [8], which ensure their potential application prospects in the field of photoemission, photodetection and field effect transistors (FETs). However, under the growing demands of material multifunction, the electronic properties of one single 2D material are far from enough [9,10]. For example, graphene, silicene and germanene, as the most promising materials, all have linear dispersion at the Fermi level at the K-point in the Brillouin zone. To our knowledge, one of the key factors for the development of 2D materials is dependent on its tunable band gap [11,12]. Thus, being a gapless semiconductor, graphene, silicene and germanene cannot be directly used in electronic optoelectronic devices [13,14]. However, if we introduce a hetero-bilayer system by stacking two single-layer materials vertically, the tunable band gaps could be realized by employing in-plane biaxial stress or changing the interlayer distance [15,16,17,18].

Recently, the heterostructures based on transition metal dichalcogenides (TMDs) [19,20], especially the WSe_2_ [21,22,23] material, have attracted extensive research due to their excellent electronic and optoelectronic properties [24,25]. For example, Ren et al. established several TMDs-based van der Waals heterostructures (MoS_2_/BP, MoSe_2_/BP, WS_2_/BP and WSe_2_/BP) with bandgaps of 1.29, 1.37, 1.22 and 1.21 eV, respectively. Among them, MoSe_2_/BP and WSe_2_/BP possess type-II band alignment with a direct band gap, which can separate the photogenerated electron–hole pairs effectively [26]. Engin Torun et al. predicted the existence of interlayer excitons (0.15 and 0.24 eV below the absorption onset of intralayer excitons) in MoS_2_/WS_2_ and MoSe_2_/WSe_2_ heterostructures, indicating that the excitonic ground states of these systems spontaneously separate the electron and the hole in different layers [27]. Si et al. also investigated the photoelectronic properties of MoS_2_/WSe_2_ heterojunction via the combination of theoretical prediction and experimental verification. They deduced that the enhancement of the photoelectric response should be attributed to the construction of the MoS_2_/WSe_2_ type-II heterostructure, which not only promotes the photogenerated electron–hole pair separation, but also suppresses their recombination [28]. Aretouli et al. found that SnSe_2_/WSe_2_ heterostructure possesses a broken gap configuration, indicating that band-to-band tunneling through an ultrathin van der Waals gap can be switched on and off easily via applying a small bias across the interface, which implies promising applications in 2D-2D vertical TFETs [29]. Thus, the heterostructures not only provide a new way to enrich the novel properties of the system but also to well preserve the electronic properties of the original freestanding two single-layer 2D components [30,31,32].

In this study, we perform ab initio calculations to investigate the electronic properties of hetero-bilayers composed of WSe_2_ monolayer and SiS_2_ monolayer (a new group of VI-IV 2D material [33]). Six possible stacking models are considered here. The geometries, relative stabilities and band structures of the considered models are discussed. Our results show that the most stable SiS_2_/WSe_2_ hetero-bilayer possesses the type-II band alignment with a narrow band gap, which contributes to the separation of electron–hole pairs. Furthermore, by applying a certain range of biaxial strain and external electric field, the band gap of the SiS_2_/WSe_2_ hetero-bilayer can be effectively tuned while maintaining the type-II band alignment. Our calculations and analysis demonstrate that the SiS_2_/WSe_2_ heterostructure may become a promising candidate material in the application of photoelectric devices.

## 2. Computational Method

To systematically investigate the structural and electronic properties of SiS_2_/WSe_2_ heterobilayers, we performed all calculations using the Vienna ab initio simulation package (VASP 5.4.1., Vienna, Austria) based on density functional theory (DFT) with the plane-wave pseudopotential methods [34,35]. The generalized gradient approximation (GGA), with the Perdew–Burke–Ernzerhof (PBE) function, was employed to describe the exchange and correlation potential [36,37]. Additionally, the hybrid Heyd–Scuseria–Eenzerhof (HSE06, Houston, TX, USA) functional was also used to obtain a more accurate bandgap [38]. In consideration of the weak van der Waals (vdW) interactions in all calculations, we used the DFT-D2 method of Grimme to correct the long-range weak vdW interlayer interactions [39]. A plane-wave kinetic energy cutoff of 500eV was adopted. The Monkhorst–Pack K-points [40] were set to 35 × 35 × 1. A large vacuum zone of 20 Å was used to make the interaction between two adjacent 2D sheets in the periodic arrangement (along the “*z*” axis) negligible. The structure relaxations were carried out until the change of the energy and the force was less than 10^−5^ and 10^−2^ eV/Å per atom, respectively.

To quantitatively characterize the stability of the heterostructure, the binding energy of SiS_2_/WSe_2_ is defined as: $E_{b}$ = $E_{SiS_{2}/WSe_{2}}-\left(E_{SiS_{2}}+E_{WSe_{2}}\right)$, where $E_{SiS_{2}/WSe_{2}}$, $E_{SiS_{2}}$ and $E_{WSe_{2}}$ represent the total energies of the SiS_2_/WSe_2_ hetero-bilayer, free-standing SiS_2_ monolayer and isolated WSe_2_ monolayer, respectively. $E_{SiS_{2}}$ is calculated by using a 1 × 1 unit cell of the SiS_2_ monolayer, and $E_{WSe_{2}}$ is calculated by using a 1 × 1 unit cell of the WSe_2_ monolayer (i.e., the size of the unit cells are the same as the supercell of the hetero-bilayer). To evaluate the interlayer electronic property and behavior of the SiS_2_/WSe_2_ heterobilayer, we also calculated the work function, defined as $\phi =\;E_{0}-E_{F}$, where $E_{0}$ and $E_{F}$ are the energy of the stationary electron in the vacuum and the Fermi level, respectively.

## 3. Results and Discussion

### 3.1. Structural Features of the Monolayer SiS_2_, WSe_2_ and SiS_2_/WSe_2_ Hetero-Bilayer

**Figure 1:**
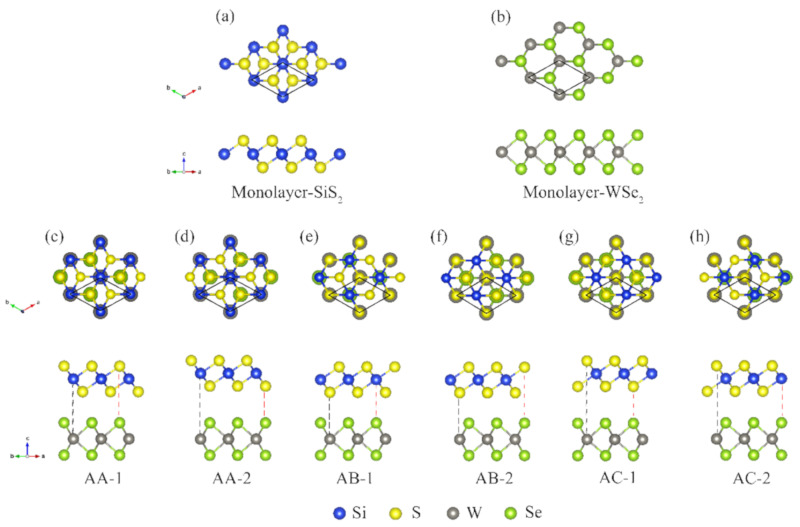
Top and side views of (**a**) monolayer SiS_2_, (**b**) monolayer WSe_2_ and (**c**–**h**) SiS_2_/WSe_2_ hetero-bilayers.

Before investigating the SiS_2_/WSe_2_ hetero-bilayer systems, we first study the electronic properties of isolated monolayer SiS_2_ and monolayer WSe_2_ (the space group of monolayer SiS_2_ and monolayer WSe_2_ are P3m1 and P63/mmc, respectively). The corresponding optimized structures of monolayer SiS_2_ and monolayer WSe_2_ are shown in Figure 1a,b. As shown in Figure 1, both of them have the same primitive cell of hexagonal structure with three atoms per unit cell. The lattice constants of SiS_2_ and WSe_2_ monolayers are calculated to be 3.30 and 3.33 Å, respectively, which agree well with previous results [33,41,42]. Compared with the hybrid systems investigated previously [21,22,23,24,25,26,27,28,29,30,31,32], such a lattice mismatch (only about 0.9%) between the SiS_2_ and WSe_2_ monolayers is very small. Thus, we have employed supercells composed of 1 × 1 unit cells of SiS_2_ monolayer and 1 × 1 unit cells of WSe_2_ monolayer in the x-y plane. To explore the possible stacking models of hetero-bilayers, we build six different stacking patterns of SiS_2_/WSe_2_ hetero-bilayers (labeled as AA-1, AA-2; AB-1, AB-2; AC-1, AC-2), as expressed in Figure 1c–h.

For AA-1 stacking, W atoms and Se atoms are located directly under the Si atoms and S (top sub-plane) atoms, respectively. For AB-1 stacking, W atoms and Se atoms are positioned just below the S atoms (bottom sub-plane) and Si atoms, respectively. For AC-1 stacking, W atoms and Se atoms are both positioned directly below the S atoms (top sub-plane and bottom sub-plane). The AA-2 (AB-2, AC-2) configuration is achieved by fixing the top layer of SiS_2_ and rotating the WSe_2_ layer of AA-1 (AB-1, AC-1) by 180 degrees with the “c” axis. The calculated binding energies for those configurations are shown in Table 1. According to our results, the binding energy of the AB-2 stacking (−197 meV) is shown to be larger than the binding energies of the other stacking models, indicating that the AB-2 model is the most stable and has the strongest bonding. These binding energies have the same order of magnitude as other typical vdW heterostructures such as the WSe_2_/BP heterostructure (−141 meV) [26] and the MoSe_2_/MoS_2_ heterostructure (−158.1 meV) [43]. In addition to the binding energy, we also investigated the bond length, the interlayer spacing (the distance between the sulfur layer of the SiS_2_ monolayer and its nearest selenium layer) and the band gaps of the hetero-bilayer systems, as shown in Table 1. Clearly, the calculated differences of lattice constants (around 2.33 Å) and bond lengths (around 2.54 Å) of Si-S and W-Se between the six hetero-bilayer models are very small. However, due to the change in relative position of atoms between the two layers, the interlayer spacings exhibit relatively larger deviations. As shown in Table 1, the AB-2 stacking configuration has the shortest interlayer distance, showing again the strongest bonding in the hetero-bilayer system. Among all six of the SiS_2_/WSe_2_ hetero-bilayers, at the PBE level, the AB-2 stacking model is the only semiconductor. Since the AB-2 stacking model is the most stable stacking pattern, we now further discuss the electronic structures of the AB-2 stacking hetero-bilayer.

### 3.2. Electronic Properties of the SiS_2_/WSe_2_ Hetero-Bilayer

**Figure 2:**
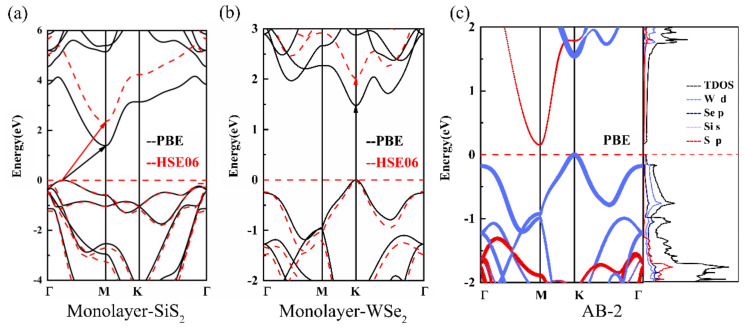
Energy band structures of (**a**) monolayer SiS_2_ and (**b**) monolayer WSe_2_. (**c**) Projected band structure and partial density of the state of AB-2 stacking SiS_2_/WSe_2_ hetero-bilayer; red and blue lines are bands contributed by SnS_2_ and WSe_2_ respectively.

Figure 2a,b demonstrates the band structures of monolayer SiS_2_ and monolayer WSe_2_ obtained by the GGA-PBE (black solid lines) and HSE06 (red dashed lines) method. It is clear that the pristine SiS_2_ monolayer displays an indirect band gap semiconductor. The band gaps of monolayer SiS_2_ obtained by the GGA-PBE and HSE06 method are 1.39 and 2.34 eV, respectively. Its valence-band maximum (VBM) is located between the high symmetry points Γ and M and the conduction-band minimum (CBM) is at the high symmetry M-point. In regard to the isolated monolayer WSe_2_, it possesses a direct band gap of 1.48 eV (PBE level) or 2.0 eV (HSE06 level) at the high symmetric K-point. These results are consistent with previous studies [33,44,45]. After the 2D materials were constructed into hetero-bilayers, the band gaps narrowed or even disappeared. However, the electronic properties of origin SiS_2_ and WSe_2_ monolayers were well preserved. As mentioned above, at PBE level, the AB-2 stacking is the most stable configuration and it is the only semiconductor among the six configurations. For the AB-2 stacking configuration, as shown in Figure 2c, the CBM and VBM of the SiS_2_ layer are both lower than those of the WSe_2_, which forms a staggered type-II indirect band alignment. To further the discussion of the electronic structures of the SiS_2_/WSe_2_ hetero-bilayer, we also investigate the density of states. The total density of states is demonstrated by the black line. The orbital occupancy of each atom is clearly demonstrated in the projected density of states (the state with a low orbital occupancy is not shown in the figure). It can be seen clearly that, near the VBM (from −1 to 0 eV), the occupied states are almost dominated by W atoms (*d* orbitals) and Se atoms (*p* orbitals). While the electronic states above the Fermi level are contributed by Si (*s* orbitals) and S atoms (*p* orbitals). These results again confirm the type II band alignment of the AB-2 SiS_2_/WSe_2_ hetero-bilayer, which can effectively separate the photogenerated holes and electrons [46,47,48,49]. Owing to the narrower band gap (0.154 and 0.738 eV obtained by GGA-PBE and HSE06, respectively), the electrons are more susceptible to being excited from VBM to CBM when the SiS_2_/WSe_2_ heterostructure is exposed to light [50]. 

On the other hand, we also calculate the effective mass of AB-2 stacking model based on the formula as follows:

(1) $$m^{*}=\;\hbar {\left(\partial^{2}E(k)/\partial k^{2}\right)}^{-1}$$

Here, ℏ is Plank’s constant, *E*(*k*) is the energy of CBM or VBM, and *k* is the wave vector. The effective mass is a key parameter to measure the mobility of carriers. Under certain conditions, the mobility μ is inversely proportional to the effective mass $m^{*}$. Our results show that the electron effective mass $m_{n}^{*}$ of CBM is 0.43 $m_{0}$ ($m_{0}$ represents the mass of a free-electron), and the hole effective mass $m_{p}^{*}$ of VBM is 0.47 $m_{0}$. A relatively small effective mass means higher carrier mobility [51]. Moreover, due to effectively separating electron–hole pairs of type-II band alignment, the lifetime of photogenerated carriers is remarkably extended.

**Figure 3:**
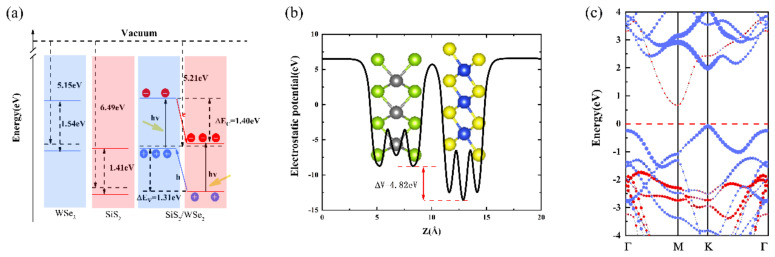
(**a**) Band alignment and (**b**) electrostatic potential of AB-2 SiS_2_/WSe_2_ hetero-bilayer obtained by GGA-PBE method. (**c**) Projected band structure of AB-2 SiS_2_/WSe_2_ hetero-bilayer obtained by the HSE06 method’ red and blue lines are bands contributed by SnS_2_ and WSe_2_ respectively.

The work function, which is crucial for evaluating the internal electronic behavior of heterostructures, is also discussed to explain the relevant charge transfer phenomenon (Figure 3a). The work functions of monolayer WSe_2_ and monolayer SiS_2_ are 5.15 and 6.49 eV respectively. Obviously, the work function of the WSe_2_ sheet is smaller than that of the SiS_2_ sheet, leading the electrons to spontaneously diffuse from WSe_2_ to the SiS_2_ layer in the SiS_2_/WSe_2_ hetero-bilayer. After the interaction between atomic layers, the Fermi level of WSe_2_ is further moved downward while the Fermi level of SiS_2_ is moved upward and finally reaches the same level, which causes the work function of the hetero-bilayer to be 5.21 eV. The same behaviors can be found in the electrostatic potential of the SiS_2_/WSe_2_ hetero-bilayer shown in Figure 3b. Due to the higher potential energy of WSe_2_, the positive charges are accumulated in the WSe_2_ layer, while the negative charges are accumulated in the SiS_2_ layer. A built-in electric field directed from WSe_2_ to SiS_2_ is thus formed on the surface of the SiS_2_/WSe_2_ hetero-bilayer, resulting in a drift movement of the internal carriers. In addition, the calculated valence band offset (VBO) $∆E_{V}$ and conduction band offset (CBO) $∆E_{C}$ between the SiS_2_ and WSe_2_ layers reach 1.31 and 1.40 eV (1.33 and 1.66 eV) obtained by GGA-PBE (HSE06) method, respectively, as shown in Figure 3a. Such a huge band offsets can remarkably prolong the lifetime of interlayer carrier (electrons and holes) and improve the efficiency of carrier separation, which plays an indispensable role in the application of optoelectronic devices. Thus, most of the photogenerated electrons are transferred from the valence band of the WSe_2_ layer to the conduction band of the SiS_2_ layer. After a few photogenerated electrons jump to the conduction band of WSe_2_ with higher energy, they will then transit to the conduction band of SiS_2_ with lower energy. The photogenerated holes transfer process of the SiS_2_/WSe_2_ hetero-bilayer functions in an opposite manner. 

In order to obtain a more accurate energy band gap, we also perform HSE06 calculations for the AB-2 stacking configuration, as shown in Figure 3c. The size of the red and blue dots respectively indicates the contribution of the SiS_2_ layer and WSe_2_ layer to the band structure. It is obvious that the the band at the CBM and VBM are contributed from the WSe_2_ layer and the SiS_2_ layer, respectively. The band gap of the SiS_2_/WSe_2_ hetero-bilayer, calculated by the HSE06 method, is 0.738 eV.

### 3.3. Effect of Biaxial Strain on Electronic Properties of SiS_2_/WSe_2_ Hetero-Bilayer

**Figure 4:**
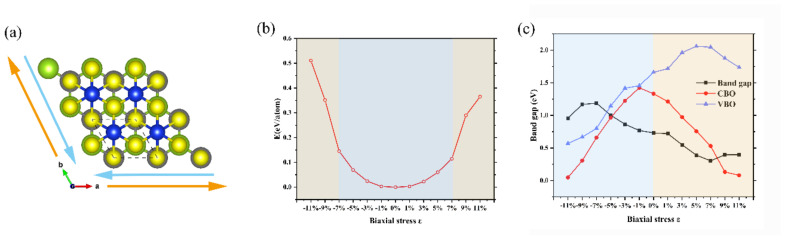
(**a**) Schematic diagram of tensile (orange arrows) and compressive (blue arrows) stresses on the SiS_2_/WSe_2_ hetero-bilayer. (**b**) Strain energy (*E*) as a function of strain of the biaxial stress ε. (**c**) Band gap and band offsets of the SiS_2_/WSe_2_ hetero-bilayer as a function of the biaxial stress ε.

As is well known, strain modulation is an effective way to alter the electronic properties of 2D vdW heterostructures [52,53,54]. In this work, we applied the in-plane biaxial strain to the SiS_2_/WSe_2_ hetero-bilayer by changing the lattice constant of the system in both the x and y directions (i.e., compressive or tensile stresses). As shown in Figure 4a, blue and orange arrows represent the compressive and tensile strain, respectively. The degree of strain (ε) is defined as follows:

(2) $$\varepsilon \;=\;\frac{a-a_{0}}{a_{0}}\;\times \;100\%$$

where *a* and $a_{0}$ correspond to the strained and unstrained lattice constants of SiS_2_/WSe_2_ hetero-bilayer, respectively. Tensile (compressive) stress is represented by ε > 0 (ε < 0). The biaxial stresses range from −11% to 11% with an interval of 2%. To avoid the structure collapse of SiS_2_/WSe_2_ hetero-bilayer, we also calculate the strain energy *E*, which is defined as follows:

(3) $$E\;=\;(E_{\mathrm{total}}\;-\;E_{0})/n$$

where $E_{\mathrm{total}}$ and $E_{0}$ represent the total energy of the strained system and the strain-free system, respectively. *N* is the number of atoms in the supercell. The results are shown in Figure 4b; the strain energy increases monotonously with increasing stress (compressive stresses: from 0 to −7%, tensile stresses: from 0 to 7%). Noteworthy is the evolution curve of the strain energy in this interval is close to the quadratic function of the strain, indicating that the stresses applied on the hetero-bilayer are within the elastic deformation limit. However, the strain energy curve begins to deviate from the original trend if the tensile (compressive) stress continues to increase, showing that the hetero-bilayer is undergoing inelastic deformation. 

We also calculate the evolution curve of the band gap and band offsets of the SiS_2_/WSe_2_ hetero-bilayer as a function of the biaxial stress *ε*, as expressed in Figure 4c. In the range of elastic deformation (the stress changes from −7% to 7%), the band gap of the SiS_2_/WSe_2_ hetero-bilayer decreases gradually with increasing tensile stress. When the applied strain exceeds the range of elastic deformation, the change trend of energy band is opposite. In regard to the band offsets of the SiS_2_/WSe_2_ hetero-bilayer, the VBO increase continuously as the strain changes from −11% to 5%, then decreases with increasing tensile stress. 

**Figure 5:**
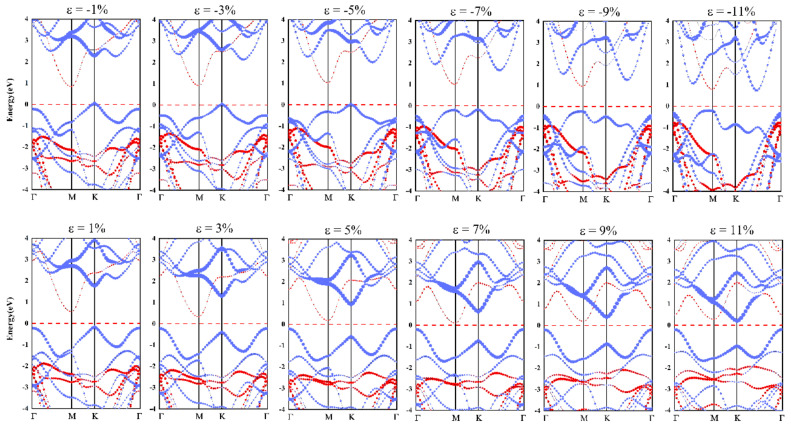
Projected band structures obtained by the HSE06 method under different in-plane biaxial stresses; red and blue lines are bands contributed by SnS_2_ and WSe_2_, respectively.

The change of band gap and band offsets of the SiS_2_/WSe_2_ hetero-bilayer can be intuitively shown in Figure 5, which is the projected band structure diagrams of the SiS_2_/WSe_2_ hetero-bilayer, obtained by the HSE06 method under different biaxial strains. The red and blue dotted lines indicate the contribution of SiS_2_ and WSe_2_, respectively. In the range of elastic deformation, the SiS_2_/WSe_2_ hetero-bilayer maintains its type-II band alignment with an indirect band gap. When the compressive stress reaches −9%, the system turns into a direct band gap semiconductor with type-II band alignment. On the other hand, the SiS_2_/WSe_2_ hetero-bilayer system changed the band alignment from type-II to type-I when the tensile stress reaches 11%, which is attractive for realizing the nano-scale multi-functional device applications.

### 3.4. Effect of Electric Field on Electronic Properties of SiS_2_/WSe_2_ Hetero-Bilayer

**Figure 6:**
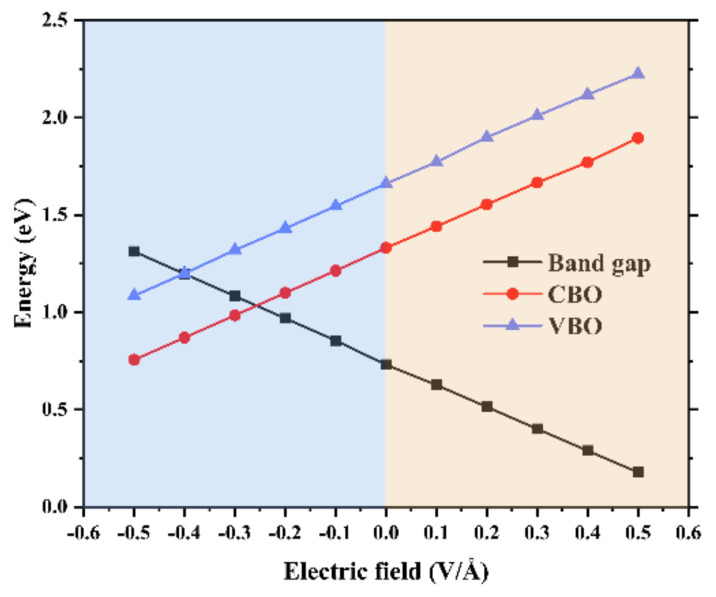
Band gap and band offsets of the SiS_2_/WSe_2_ hetero-bilayer as a function of the external electric field.

**Figure 7:**
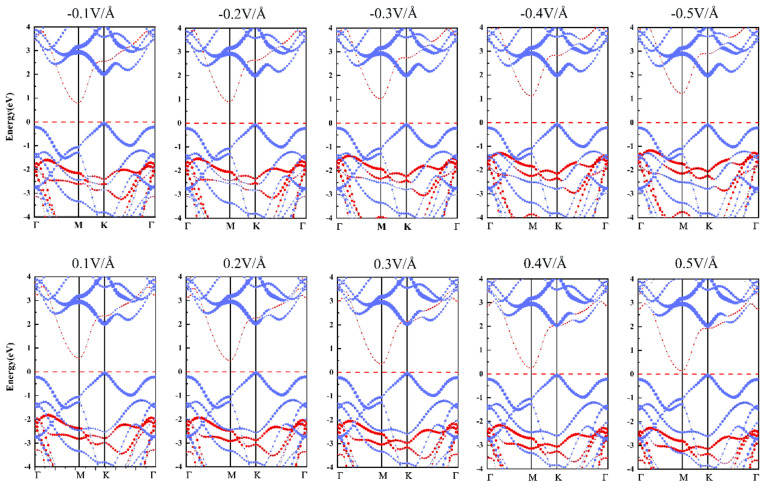
Projected band structures of the SiS_2_/WSe_2_ hetero-bilayers under different external electric fields obtained by the HSE06 method; red and blue lines are bands contributed by SnS_2_ and WSe_2_ respectively.

Applying an external electric field (*E_ext_*) has proven to be an effective method to tune the band gap [55,56]. In this section, we apply a vertical electric field (*E_ext_*) along the z direction to the SiS_2_/WSe_2_ hetero-bilayer. The direction from the SiS_2_ layer to the WSe_2_ layer is defined as the positive direction of the *E_ext_*, which is opposite to the direction of the *E_int_* in the hetero-bilayer. The value of the band gap gradually increases with increasing negative *E_ext_*, and reduces continuously with the increasing positive *E_ext_*, as shown in Figure 6. The band gap as a function of the external electric field shows a trend of completely linear decrease, while the changes of VBO and CBO show a linear increase trend. The projected band structures of the SiS_2_/WSe_2_ hetero-bilayer under various *E_ext_* are displayed in Figure 7. We find that the hetero-bilayer system could retain type-II band alignment features in the range of −0.1 V/Å to 0.5 V/Å for the external E-field, indicating that the *E_ext_* has little influence on the variations of the band structure of the systems. This is essential for the future application of the SiS_2_/WSe_2_ hetero-bilayer-based electronic devices, such as the field-effect transistor.

## 4. Conclusions

In summary, the structural and electronic properties of the SiS_2_/WSe_2_ hetero-bilayers are investigated in detail through first principles calculations. Our results show that the SiS_2_/WSe_2_ hetero-bilayer is an indirect band gap semiconductor (0.154 and 0.738 eV obtained by GGA-PBE and HSE06, respectively) with an intrinsic type-II band alignment. Meanwhile, the heterostructure perfectly retains the electronic properties of the pristine 2D monolayer components. Moreover, the SiS_2_/WSe_2_ hetero-bilayers have been shown to possess a relatively low effective mass, which enhances carrier mobility of the heterostructure. The type-II band alignment, narrow band gap, together with low effective mass conduce effective separation of photogenerated carriers, which is promising for application in optoelectronic devices. On the other hand, under biaxial strain, the heterostructure can withstand the biaxial strain from −7% (compressive) to 7% (tensile) while maintaining the type-II band alignment. Moreover, through changing the effective electric field crossing the interface of the heterostructure, the band gap and band offset of the SiS_2_/ WSe_2_ hetero-bilayers can be effectively modulated by applying the external electric field. Our results offer promising alternatives for the engineering of two dimensional material-based optoelectronic nanodevices.

## Figures and Tables

**Table 1:** The optimized structural parameters of SiS_2_/WSe_2_ heterostructures with different configurations, including the binding energy ($E_{b}$ ), lattice constant (*a*), bond length ($d_{\mathrm{Si}-S}$, $d_{W-\mathrm{Se}}$ ), the interlayer spacing (*s*), and the band gap (*Eg*) of the system obtained by GGA-PBE and hybrid HSE06.

Stacking Mode	$E_{b}\;(\mathrm{meV})$	*a* (Å)	$d_{\mathrm{Si}-S}\;(Å)$	$d_{W-\mathrm{Se}}\;(Å)$	*s* (Å)	$E_{g}^{\mathrm{PBE}}\;(\mathrm{eV})$	$E_{g}^{\mathrm{HSE}06}\;(\mathrm{eV})$
AA-1	−182.5	3.315	2.325	2.541	3.192	metal	/
AA-2	−125.1	3.315	2.326	2.542	3.784	metal	/
AB-1	−194.2	3.315	2.325	2.540	3.144	metal	/
AB-2	−197.0	3.315	2.324	2.541	3.125	0.154	0.738
AC-1	−125.0	3.314	2.325	2.541	3.788	metal	/
AC-2	−175.0	3.317	2.325	2.542	3.234	metal	/
